# Low frequency mitochondrial DNA heteroplasmy SNPs in blood, retina, and [RPE+choroid] of age-related macular degeneration subjects

**DOI:** 10.1371/journal.pone.0246114

**Published:** 2021-01-29

**Authors:** Shari R. Atilano, Nitin Udar, Timothy A. Satalich, Viraat Udar, Marilyn Chwa, M. Cristina Kenney

**Affiliations:** 1 Gavin Herbert Eye Institute, University of California Irvine, Irvine, CA, United States of America; 2 Institute for Mathematical Behavioral Science, University of California Irvine, Irvine, CA, United States of America; 3 Department of Pathology and Laboratory Medicine, University of California Irvine, Irvine, CA, United States of America; University of Florida, UNITED STATES

## Abstract

**Purpose:**

Mitochondrial (mt) DNA damage is associated with age-related macular degeneration (AMD) and other human aging diseases. This study was designed to quantify and characterize mtDNA low-frequency heteroplasmy single nucleotide polymorphisms (SNPs) of three different tissues isolated from AMD subjects using Next Generation Sequencing (NGS) technology.

**Methods:**

DNA was extracted from neural retina, [RPE+choroid] and blood from three deceased age-related macular degeneration (AMD) subjects. Entire mitochondrial genomes were analyzed for low-frequency heteroplasmy SNPs using NGS technology that independently sequenced both mtDNA strands. This deep sequencing method (average sequencing depth of 30,000; range 1,000–100,000) can accurately differentiate low-frequency heteroplasmy SNPs from DNA modification artifacts. Twenty-three ‘hot-spot’ heteroplasmy mtDNA SNPs were analyzed in 222 additional blood samples.

**Results:**

Germline homoplasmy SNPs that defined mtDNA haplogroups were consistent in the three tissues of each subject. Analyses of SNPs with <40% heteroplasmy revealed the blood had significantly greater numbers of heteroplasmy SNPs than retina alone (p≤0.05) or retina+choroid combined (p = 0.008). Twenty-three ‘hot-spot’ mtDNA heteroplasmy SNPs were present, with three being non-synonymous (amino acid change). Four ‘hot-spot’ heteroplasmy SNPs (m.1120C>T, m.1284T>C, m.1556C>T, m.7256C>T) were found in additional samples (n = 222). Five heteroplasmy SNPs (m.4104A>G, m.5320C>T, m.5471G>A, m.5474A>G, m.5498A>G) declined with age. Two heteroplasmy SNPs (m.13095T>C, m.13105A>G) increased in AMD compared to Normal samples. In the heteroplasmy SNPs, very few transversion mutations (purine to pyrimidine or vice versa, associated with oxidative damage) were found and the majority were transition changes (purine to purine or pyrimidine to pyrimidine, associated with replication errors).

**Conclusion:**

Within an individual, the blood, retina and [RPE+choroid] contained identical homoplasmy SNPs representing inherited germline mtDNA haplogroup. NGS methodology showed significantly more mtDNA heteroplasmy SNPs in blood compared to retina and [RPE+choroid], suggesting the latter tissues have substantial protection. Significantly higher heteroplasmy levels of m.13095T>C and m.13105A>G may represent potential AMD biomarkers. Finally, high levels of transition mutations suggest that accumulation of heteroplasmic SNPs may occur through replication errors rather than oxidative damage.

## Introduction

Mitochondria (mt) have their own DNA that is inherited through the maternal lineage and the mtDNA haplogroups have been used to classify the ancestral distribution of human populations. The human mtDNA forms a closed circle of double stranded DNA, with 16,569 nucleotide pairs, comprised of two strands that are differentiated by their nucleotide content. The heavy strand is guanine rich and encodes for 28 genes while the light strand is cytosine rich and encodes for 9 genes. Unlike the nuclear genome, mtDNA contains a unique non-coding Control Region and no introns. The non-coding mtDNA Dloop has within it the 1121 nucleotide control region that is important for replication and transcription. The coding region of mtDNA codes for 37 genes including 13 protein subunits essential for oxidative phosphorylation (OXPHOS), 2 ribosomal RNAs and 22 transfer RNAs [1–3]. The majority of mitochondrial proteins (~1500–2000) are encoded by nuclear DNA (nDNA) and imported into the mitochondria, where they participate in energy production [4, 5].

While there is only a single DNA copy of the nuclear genome within each cell, there can be multiple copies of mtDNA, since depending upon the level of metabolic activity, a cell can have hundreds of mitochondria, each of which possesses 1 to 10 copies of mtDNA. Cells can have homoplasmic mtDNA, indicating that all mtDNA copies are identical [6]. However, with increased oxidative stress associated with aging, the mtDNA can be damaged and as there are poor repair systems for mtDNA, these sporadically mutated SNPs accumulate within the cell. Heteroplasmy occurs when there is a mixture of the damaged, mutant mtDNA along with the non-mutated, undamaged (wildtype) mtDNA within the same cell. Upon cell division, the mitochondria with heteroplasmic mtDNA can be randomly or stochastically distributed to the daughter cells [7–11] so that some daughter cells may receive high burden of mitochondria with heteroplasmic mtDNA while others do not. This is important because only relatively low levels of heteroplasmy can be tolerated by cells before abnormal function and disease can occur. With Sanger sequencing, the lowest threshold of heteroplasmy detection is approximately 30%. To date there have not been readily available techniques to perform in-depth sequencing of the mtDNA that would yield identification of heteroplasmy at percentage frequency ranges below 5%. Most studies use only the mitochondrial haplogroup variations for determining disease associations. What is lacking in most studies is the determination of variations throughout the entire mitochondrial genome (16,569 bps). The Next Generation Sequencing (NGS) technologies are currently performing whole genome sequencing; however accurately identifying low frequency mitochondrial variations has been a challenge. DNA modification occurs after DNA extraction and artifacts can be interpreted as low frequency mutations. The dual strand approach to NGS, as utilized in this study, is a novel approach to studying somatic mutations in mitochondrial DNA.

Age-related macular degeneration (AMD) is the most common cause of vision loss in the elderly in developed countries. Mitochondrial dysfunction and damage are clearly associated with AMD [12–21]. Cybrid studies have shown that in addition to influencing energy pathways, the mtDNA variants mediate expression of nuclear genes related to complement, inflammation, apoptosis, autophagy, methylation and cell signaling [22–26]. This is significant because the complement, apoptosis and autophagy pathways have been shown both in vitro and in vivo to be involved in the pathogenesis of AMD [27–30]. Transmission electron microscopy and immunohistochemistry of the retinal pigment epithelial (RPE) cells from AMD eyes has shown that mitochondria are damaged, fragmented and disrupted [20, 21, 31]. The RPE cells have high numbers of mtDNA lesions and fragmentations that can be linked to the severity of AMD [16]. Interestingly, the mtDNA damage was found in RPE cells and not in the neural retinal cells [32].

The retina has one of the highest rates of metabolic activity of any tissue in the body and thereby requires high levels of mitochondrial OXPHOS. The mitochondria are one of the major endogenous producers of reactive oxygen species (ROS), which can then damage proteins, lipids and DNA. Furthermore, the levels of destruction increase with aging so that oxidative damage to the mtDNA would be highest in older subjects compared to younger subjects. Based upon these findings, our study proposed 3 hypotheses (**a**) within a single individual the accumulation of damage-associated heteroplasmic SNPs would be greatest in the retinal mtDNA compared to peripheral blood; (**b**) the majority of acquired (somatic) heteroplasmy mutations would result from oxidative stress-associated transversion changes (purine to pyrimidine and vice versa); and (**c**) ‘hotspots’ for heteroplasmy SNPs could be identified. To test these hypotheses, we use Next Generation Sequencing (NGS) technology to characterize similarities and differences of mtDNA signatures in three different tissues (blood, [RPE+choroid] and retina) from three AMD individuals and compare the ‘hotspot’ heteroplasmy SNP with an additional 222 subjects.

## Methods

### Tissue collection

Human Eye Globes and blood from three AMD subjects were collected from the San Diego Eye Bank and globes were dissected to separate the [RPE+choroid] and retina tissues. The DNA was extracted from blood, [RPE+choroid] and retina (neural retina). The diseased specimens had a clinical diagnosis and medical history of AMD that had significantly impaired vision. Two of the subjects had wet AMD while the third had dry AMD (**Table 1**). A board-certified ophthalmologist verified AMD disease in the macular region by using the Minnesota Grading System of Eye Bank Eyes. All clinical investigations and protocols were conducted according to the principles of the Declaration of Helsinki and approved by written consent by the University of California, Irvine Office of Research Institutional Review Board (IRB#2003–3131).

**Table 1:** Description of the AMD subjects.

	Age	Gender	AMD	Haplogroup
**Subject 1**	93	F	DRY	I1a1b
**Subject 2**	88	F	WET	H7b
**Subject 3**	90	M	WET	U2e2a1

### Extraction of total DNA from blood, retinal, and [RPE+choroid] tissues

The total DNA was isolated from 10 mls venous blood samples (Puregene; Gentra, Minneapolis, MN). The total DNA was extracted from frozen retinal tissue and [RPE+choroid] by methods published previously [15]. For the extraction of [RPE+choroid] DNA, no centrifugation steps were performed because of the co-precipitation of DNA with melanin, a known inhibitor of Polymerase Chain Reaction (PCR). Instead, after the precipitation of DNA in ammonium acetate, the DNA precipitate was removed and dip washed in 70% and 100% ethanol. Then the DNA was dried and resuspended in Tris-EDTA (TE) [15]. For analyses of the 23 heteroplasmy mtDNA ‘hot-spots’, the NGS results for an additional 222 subjects were analyzed: Younger normal subjects (Young-NL, n = 83, age range 18–55 yo); Old Non-AMD subjects (Old-NL, n = 66, age range 56–93 yo); and AMD subjects (Old-AMD, n = 73, age range 59–94 yo, 18 Dry AMD, 55 Wet AMD) (S1 Table).

### Sequencing of mtDNA from AMD tissue samples

Our NGS technology sequenced both strands of mtDNA independently in both directions (forward and reverse), which allowed us to quantify the haplogroup-defining single nucleotide polymorphisms (SNPs), private SNPs (not defining haplogroups), unique SNPs (SNPs not found in www.MitoMap.org or any other database) and low frequency heteroplasmy SNPs across the entire mitochondrial genome. A high degree of confidence was achieved with our method because the dual strand sequencing in both directions gave us a depth of sequencing that ranged from 1,000 to 100,000 and allowed us to conclude with a high degree of confidence that SNP changes were indeed heteroplasmy and not artifacts.

Depending on the method used, mtDNA variations can be further classified as homoplasmic in which all of the mitochondria are identical at or near 100% minor allele frequency (MAF) or heteroplasmic in which there are multiple mtDNA variations in the cell. The success in determining these variations has been limited by technology that could not effectively sequence heteroplasmic variations at less than 30% frequency. Since each mitochondrion in a single cell can have different mtDNA, it has been difficult to estimate the total mitochondrial variation in a population of cells (e.g. blood or tissue). In order to determine the heteroplasmic nature of mtDNA variants at low frequency across the entire mitochondrial genome, we have developed a novel mtDNA sequencing strategy that can accurately identify low frequency (less than 5%) mtDNA variations from DNA modification artifacts. This novel approach includes sequencing both strands of mtDNA independently using the NGS technology. We then use a bioinformatics approach to determine if the variants were identified on one or both strands of mtDNA. If the variant is present on both strands, it is considered a ‘true variant’. If the variant is present on only one strand (as in the case of a DNA modification), then it is considered an artifact. This approach has allowed us to distinguish true variants from artifacts down to 1% MAF.

Primers across the entire human mitochondrial genome were designed to eliminate the nuclear mitochondrial pseudogenes (NUMT-pseudogenes). Each primer was tested against a nuclear DNA standard and if there was amplification, then that primer was discarded and not used. A total of 171 primer pairs were designed that overlapped each other to capture the intervening sequences. A total of 100 ng of DNA per sample was used to construct NGS libraries using the TruSeq Custom Amplicon kit (Illumina, San Diego, USA). Two independent sets of primer pools were synthesized. The two primer pools had primers that were complementary. The two primer pools would interrogate the two strands of the mitochondrial genome independently. The two independent libraries had 48 samples each. NGS sequencing was performed on a HiSeq instrument (Illumina) and the somatic variant analyses were carried out using the variant caller–Pisces [33]. Variant calls from each pool were combined to get a list of ‘true variants’, which are defined as variants present on both strands of the mtDNA. The ‘true variant’ list was used for final haplogroup analyses using Haplogrep (www.haplogrep.uibk.ac.at). Variants present on only one strand may represent mtDNA modification events or other artifacts especially at low frequency and therefore were eliminated from the final analysis. This method is capable of deep sequencing (average sequencing depth of 30,000; range 1,000 to 100,000) and accurately differentiates low frequency mtDNA heteroplasmy SNPs from mtDNA modification artifacts.

The amino acid changes and association with diseases resulting from some of the SNPs variants were verified using www.MitoMap.org [34, 35]. In some cases, the website www.Phylotree.org [36] was used to verify the positions of specific SNPs within the haplogroups and also to determine if the SNPs were elsewhere in the entire mtDNA tree Build 17. The rs (Reference SNP cluster ID) numbers were identified using www.ncbi.nlm.nih.gov/snp. The website www.hmtvar.uniba.it, a database with over 40K human mtDNA variants [37], was utilized to search for specific variants using the query tab and inputting the SNP into the position field to determine mutation, amino acid change (if any), and locus, as well as the link to external resources such as dbSNP (SNP Database) for rs numbers. All SNPs identified had a quality score of 100 (A Phred-scaled quality score assigned by the variant caller) and PASSed all of the filters. Sequencing data can be found at www.ncbi.nlm.gov under Sequence Read Archive ID PRJNA688521.

### Statistical analyses

Statistical analysis of the data was performed by ANOVA (GraphPad Prism, version 5.0). Newman-Keuls multiple-comparison or the two-tailed *t*-tests were used to compare the data within each experiment. Some data were analyzed using the 2x2 contingency table with Fisher’s exact test and two-tailed *t*-tests (MEDCALC, Software). P < 0.05 was considered statistically significant. Error bars in the graphs represent standard error of the mean (SEM).

## Results

### Total numbers of SNPs

**Fig 1:**
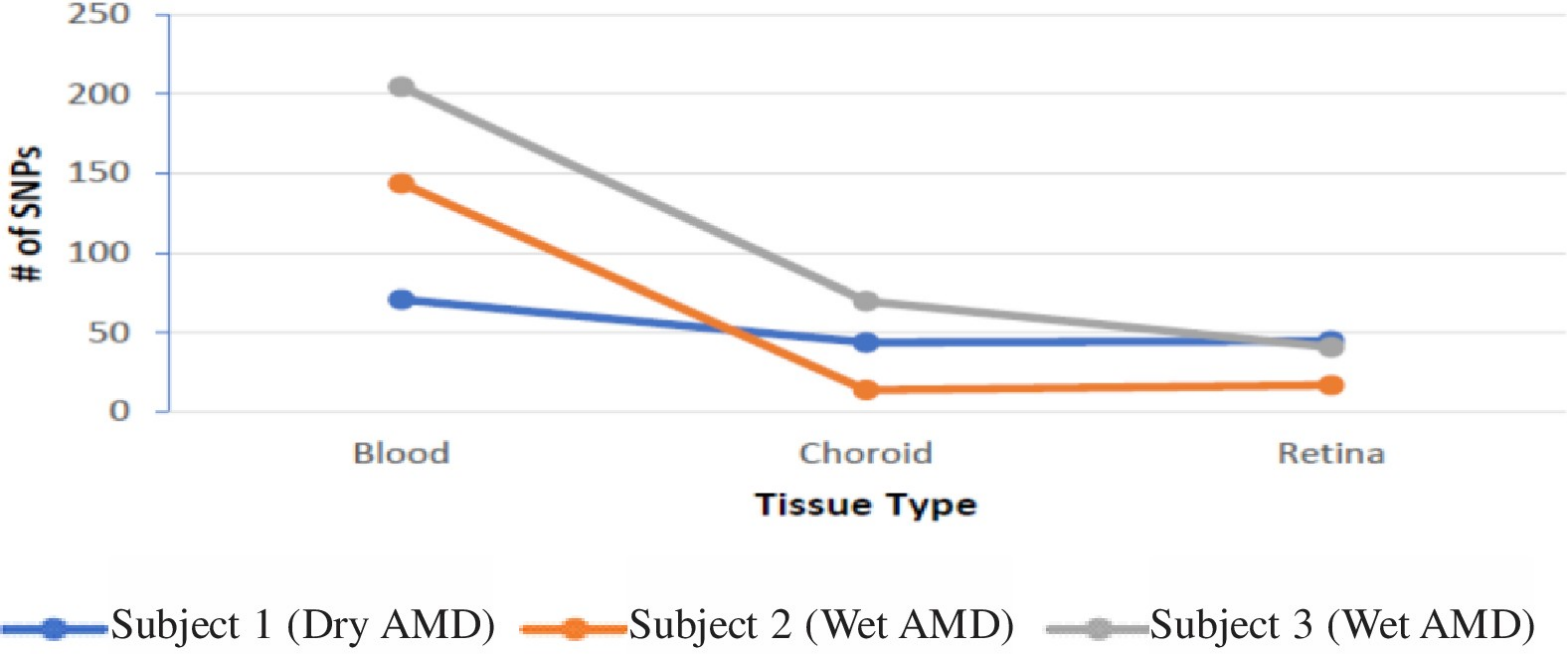
Number of SNPs for subjects and tissue types.

The NGS technique identified haplogroup defining SNPs, Private SNPs, unique SNPs and heteroplasmy SNPs. The total numbers of SNPs varied considerably within the different individuals. The total SNP number for the blood, [RPE+choroid] and retina for Subject #1 was 160, while Subject #2 had 175 SNPs and Subject #3 had 316 SNPs (**Table 2 and Fig 1**). These results show that within each Subject the numbers of SNPs in the blood were higher compared to those in either the [RPE+choroid] or retina mtDNA. Sequencing data can be found at www.ncbi.nlm.gov under Sequence Read Archive ID PRJNA688521.

**Table 2:** Total numbers of SNPs: Combining haplogroup defining SNPs, private SNPs, unique SNPs and heteroplasmy SNPs.

	Blood	[RPE+choroid]	Retina	Total
**Subject 1**	71 (44.4%)	44 (27.5%)	45 (28.1%)	160
**Subject 2**	144 (82.3%	12 (6.9%)	17 (9.7%)	175
**Subject 3**	205 (64.9%)	70 (22.2%)	41 (13.0%)	316
**Total**	420 (64.5%)	128 (19.7%)	103 (15.8%)	651

### Germline homoplasmy

The total number of the germline homoplasmy SNPs found in the different tissues (blood, [RPE+choroid] or retina) from Subjects #1, #2 and #3 was analyzed (**Table 3**). As expected for tissues from the same individual, the homoplasmy SNPs found within the blood, [RPE+choroid] and retina were consistent for each subject; Subject #1, 30:30:30; Subject #2, 3:3:3; Subject #3, 32;32;32. **Table 3** shows the frequency for homoplasmy SNPs in the subject’s tissues ranges from 89.53% (m.6045C>T) to 99.9% (m.10398A>G). When haplogroup defining SNPs were analyzed, Subject #1 was identified as mtDNA haplogroup I1a1b, Subject #2 was H7b and Subject #3 was U2e2a1. There were no SNPs that are private (non-haplogroup defining) or unique (not listed in www.mitomap.org) but rather all homoplasmy SNPs were those that defined the individual’s maternally inherited haplogroup. In summary of the germline studies, the NGS method consistently identified in all three tissues those SNPs that allowed comprehensive subset analyses of the haplogroups.

**Table 3:** List of homoplasmy SNPs found in different tissues and subjects.

Change	% Frequency			% Frequency			% Frequency		
	Subject 1			Subject 2			Subject 3		
Total	Homoplasmy SNPs = 30			Homoplasmy SNPs = 3			Homoplasmy SNPs = 32		
	Blood	[RPE+choroid]	Retina	Blood	[RPE+choroid]	Retina	Blood	[RPE+choroid]	Retina
73A>G	**99.29%**	**99.73%**	**99.81%**	**73A = H Haplogroup Defining (R0)**			**99.68%**	**99.79%**	**99.81%**
152T>C	**-**	**-**	**-**	**-**	**-**	**-**	**99.69%**	**99.78%**	**99.76%**
195T>C	**-**	**-**	**-**	**-**	**-**	**-**	**99.57%**	**99.68%**	**99.73%**
199T>C	**98.25%**	**98.90%**	**99.29%**	**-**	**-**	**-**	**-**	**-**	**-**
203G>A	**98.51%**	**99.16%**	**99.54%**	**-**	**-**	**-**	**-**	**-**	**-**
204T>C	**99.04%**	**98.13%**	**99.01%**	**-**	**-**	**-**	**-**	**-**	**-**
217T>C	**-**	**-**	**-**	**-**	**-**	**-**	**99.53%**	**98.25%**	**97.01%**
263A>G	**-**	**-**	**-**	**99.81%**	**99.82%**	**99.82%**	**99.77%**	**99.75%**	**99.80%**
508A>G	**-**	**-**	**-**	**-**	**-**	**-**	**99.70%**	**99.70%**	**99.79%**
1719G>A	**99.43%**	**99.51%**	**99.70%**	**-**	**-**	**-**	**-**	**-**	**-**
1811A>G	**-**	**-**	**-**	**-**	**-**	**-**	**97.82%**	**99.12%**	**99.44%**
2706A>G	**99.68%**	**99.79%**	**99.84%**	**2706A = H Haplogroup Defining**			**99.77%**	**99.76%**	**99.78%**
3447A>G	**99.38%**	**99.70%**	**99.79%**	**-**	**-**	**-**	**-**	**-**	**-**
3720A>G	**-**	**-**	**-**	**-**	**-**	**-**	**99.75%**	**99.71%**	**99.81%**
3849G>A	**-**	**-**	**-**	**-**	**-**	**-**	**99.70%**	**99.73%**	**99.73%**
3990C>T	**98.66%**	**99.17%**	**99.03%**	**-**	**-**	**-**	**-**	**-**	**-**
4529A>T	**98.60%**	**99.12%**	**99.12%**	**-**	**-**	**-**	**-**	**-**	**-**
4553T>C	**-**	**-**	**-**	**-**	**-**	**-**	**90.03%**	**98.53%**	**98.96%**
4736T>C	**-**	**-**	**-**	**-**	**-**	**-**	**99.81%**	**99.74%**	**99.81%**
4793A>G	**-**	**-**	**-**	**97.73%**	**99.37%**	**99.08%**	**-**	**-**	**-**
5348C>T	**-**	**-**	**-**	**97.72%**	**99.36%**	**99.05%**	**-**	**-**	**-**
5390A>G	**-**	**-**	**-**	**-**	**-**	**-**	**92.42%**	**98.88%**	**99.22%**
5426T>C	**-**	**-**	**-**	**-**	**-**	**-**	**99.68%**	**99.74%**	**99.77%**
6045C>T	**-**	**-**	**-**	**-**	**-**	**-**	**89.53%**	**98.48%**	**98.98%**
6152T>C	**-**	**-**	**-**	**-**	**-**	**-**	**89.66%**	**98.62%**	**98.83%**
6734G>A	**98.38%**	**99.37%**	**99.38%**	**-**	**-**	**-**	**-**	**-**	**-**
7028C>T	**99.13%**	**99.45%**	**99.60%**	**7028C = H Haplogroup Defining**			**95.95%**	**99.25%**	**99.41%**
8251G>A	**98.38%**	**99.29%**	**99.26%**	**-**	**-**	**-**	**-**	**-**	**-**
8473T>C	**-**	**-**	**-**	**-**	**-**	**-**	**90.96%**	**98.73%**	**99.18%**
9947G>A	**99.48%**	**99.63%**	**99.70%**	**-**	**-**	**-**	**-**	**-**	**-**
10034T>C	**99.73%**	**99.76%**	**99.84%**	**-**	**-**	**-**	**-**	**-**	**-**
10238T>C	**99.70%**	**99.71%**	**99.84%**	**-**	**-**	**-**	**-**	**-**	**-**
10398A>G	**99.68%**	**99.86%**	**99.90%**	**-**	**-**	**-**	**-**	**-**	**-**
10876A>G	**-**	**-**	**-**	**-**	**-**	**-**	**99.05%**	**99.60%**	**99.75%**
10915T>C	**99.73%**	**99.75%**	**99.82%**	**-**	**-**	**-**	**-**	**-**	**-**
11467A>G	**-**	**-**	**-**	**-**	**-**	**-**	**98.78%**	**99.66%**	**99.75%**
11719G>A	**99.37%**	**99.66%**	**99.71%**	**11719G = H Haplogroup Defining (R0)**			**99.63%**	**99.51%**	**99.66%**
12308A>G	**-**	**-**	**-**	**-**	**-**	**-**	**99.74%**	**99.77%**	**99.81%**
12372G>A	**-**	**-**	**-**	**-**	**-**	**-**	**99.68%**	**99.63%**	**99.71%**
12501G>A	**97.98%**	**99.16%**	**99.16%**	**-**	**-**	**-**	**-**	**-**	**-**
12557C>T	**-**	**-**	**-**	**-**	**-**	**-**	**89.93%**	**98.32%**	**98.91%**
12705C>T	**99.12%**	**99.59%**	**99.71%**	**-**	**-**	**-**	**-**	**-**	**-**
13020T>C	**-**	**-**	**-**	**-**	**-**	**-**	**89.55%**	**98.46%**	**98.92%**
13734T>C	**-**	**-**	**-**	**-**	**-**	**-**	**99.75%**	**99.70%**	**99.80%**
13780A>G	**99.45%**	**99.58%**	**99.67%**	**-**	**-**	**-**	**-**	**-**	**-**
14182T>C	**99.72%**	**99.72%**	**99.75%**	**-**	**-**	**-**	**-**	**-**	**-**
14766C>T	**99.62%**	**99.76%**	**99.79%**	**14766C = H Haplogroup Defining (HV)**			**99.71%**	**99.73%**	**99.75%**
15043G>A	**99.40%**	**99.60%**	**99.67%**	**-**	**-**	**-**	**-**	**-**	**-**
15907A>G	**-**	**-**	**-**	**-**	**-**	**-**	**99.76%**	**99.78%**	**99.81%**
15924A>G	**99.74%**	**99.75%**	**99.77%**	**-**	**-**	**-**	**-**	**-**	**-**
16051A>G	**-**	**-**	**-**	**-**	**-**	**-**	**99.65%**	**99.57%**	**99.73%**
16129G>A	**99.13%**	**95.51%**	**92.08%**	**-**	**-**	**-**	**-**	**-**	**-**
16129G>C	**-**	**-**	**-**	**-**	**-**	**-**	**99.56%**	**99.58%**	**99.58%**
16172T>C	**99.64%**	**99.63%**	**99.79%**	**-**	**-**	**-**	**-**	**-**	**-**
16189T>C	**-**	**-**	**-**	**-**	**-**	**-**	**97.23%**	**96.55%**	**97.61%**
16223C>T	**99.28%**	**99.45%**	**99.66%**	**-**	**-**	**-**	**-**	**-**	**-**
16311T>C	**99.64%**	**99.35%**	**99.70%**	**-**	**-**	**-**	**-**	**-**	**-**
16362T>C	**-**	**-**	**-**	**-**	**-**	**-**	**99.54%**	**99.60%**	**99.70%**
16391G>A	**99.19%**	**99.22%**	**99.48%**	**-**	**-**	**-**	**-**	**-**	**-**

These are haplogroup defining SNPs.

**-** No Single Nucleotide Polymorphism found.

### Heteroplasmy SNP patterns in the entire mtDNA

**Fig 2:**
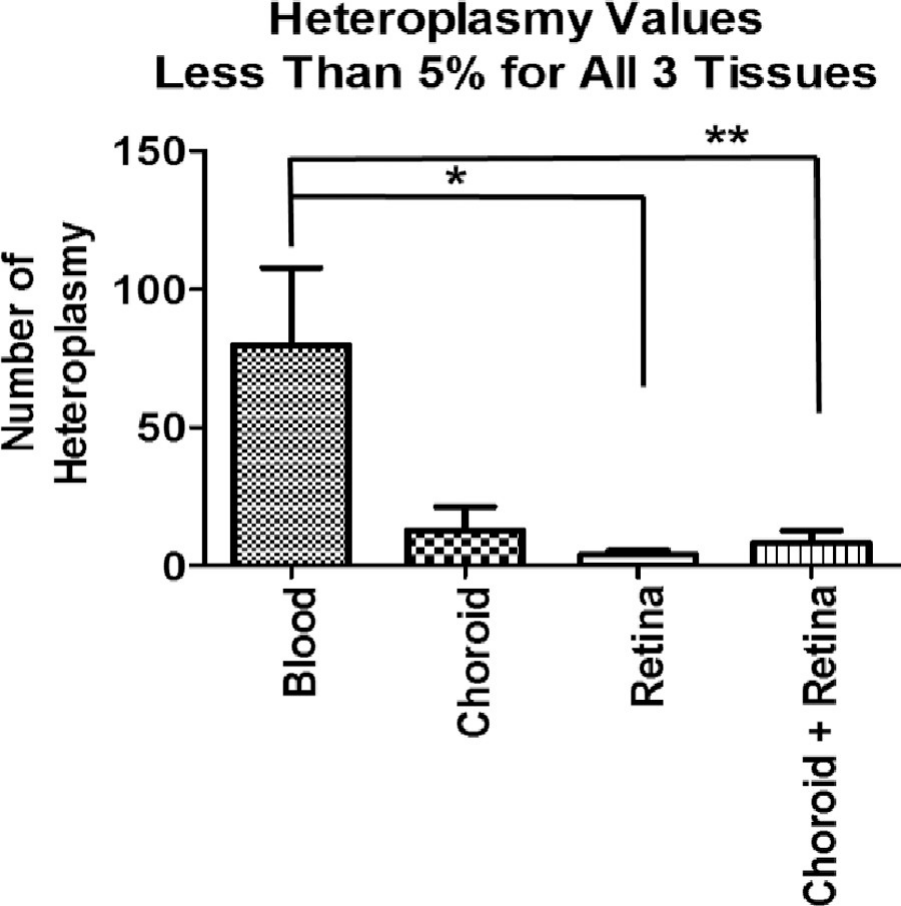
Number of heteroplasmy SNPs found in blood, choroid, and retina.

The total numbers of heteroplasmy SNPs found in the different tissues (blood, [RPE+choroid] or retina) from Subjects #1, #2 and #3 were analyzed. The numbers of SNPs with less than 40% frequency found within the blood, [RPE+choroid] and retina varied between the different subjects; Subject #1, 35:8:9; Subject #2, 135:5:8; Subject #3, 165:32;3 (**Table 4**). The numbers of heteroplasmy SNPs found in blood (83.8%) was significantly higher than for the retina (5%) alone (95% higher, P < 0.05) or the retina + [RPE+choroid] (11.3%), (89% higher, P < 0.01) (**Fig 2**). **Table 5** shows the specific heteroplasmy SNPs and their percentage frequency in each sample. The NGS methods can accurately identify low frequency heteroplasmy in the ranges from 1.16% (m.6023G>A and m.1284T>C) with the highest at 26.73% (m.16182A>C). There were only two heteroplasmy SNPs in the mid-50’s range– m.16183A>C (58.92% to 60.43%) and m.1661A>G (50.55%). The m.1661A>G was not found in any of the other 222 subjects analyzed. However, m.16183A>C was found in 14/222 subjects (2 with diabetic retinopathy; 5 with wet AMD; 1 with dry AMD and 6 normals).

**Table 4:** Heteroplasmy SNPs found in different tissues and subjects (see Table 5 for complete list).

	Blood Heteroplasmy SNPs	[RPE+choroid] Heteroplasmy SNPs	Retina Heteroplasmy SNPs	Total per subject
**Subject 1**	35 (67.3%)	8 (15.4%	9 (17.3%)	52
**Subject 2**	135 (91.2%)	5 (3.4%)	8 (5.4%)	148
**Subject 3**	165 (82.5%)	32 (16.0%)	3 (1.5%)	200
**TOTAL per tissue**	335 (83.8%)	45 (11.3%)	20 (5.0%)	

**Table 5:** List of heteroplasmy SNPs found in different tissues and subjects.

Change	% Frequency Subject 1			% Frequency Subject 2			% Frequency Subject 3		
	Blood	RPE+choroid	Retina	Blood	RPE+choroid	Retina	Blood	RPE+choroid	Retina
**Total Heteroplasmy**	**35**	**8**	**9**	**135**	**5**	**8**	**165**	**32**	**3**
64C>T	-	-	-	-	1.21%	-	-	-	-
72T>C	-	-	-	-	5.22%	8.62%	-	-	-
76C>T	-	3.60%	1.80%	-	-	-	-	-	-
189A>G	-	-	-	-	-	-	-	1.41%	1.56%
309C>T	4.86%	2.90%	3.37%	3.36%	3.47%	3.40%	-	-	-
310T>C	10.75%	8.91%	8.60%	8.80%	9.13%	9.02%	-	3.55%	-
328A>G	11.60%	19.38%	8.04%	-	-	-	-	-	-
567A>C	3.36%	-	-	-	-	-	-	-	-
813A>G	-	-	-	-	-	-	7.56%	-	-
825T>A	-	-	-	-	-	-	7.50%	-	-
868C>T	1.44%	-	-	-	-	-	-	-	-
1039A>G	-	-	-	-	-	-	1.19%	-	-
1040T>C	-	-	-	-	-	-	1.14%	-	-
1106C>T	-	-	-	1.72%	-	-	4.16%	-	-
1120C>T	1.67%	-	-	3.23%	-	1.24%	8.92%	1.40%	-
1123C>G	-	-	-	-	-	-	3.66%	-	-
1556C>T	2.14%	-	-	3.68%	-	1.38%	10.52%	1.69%	-
1267T>C	-	-	-	2.09%	-	-	6.55%	-	-
1284T>C	1.90%	-	-	2.73%	-	1.16%	8.20%	-	-
1291T>C	-	-	-	1.98%	-	-	6.49%	-	-
1292A>G	1.81%	-	-	2.69%	-	-	8.03%	-	-
1520T>C	-	-	-	1.88%	-	-	5.40%	-	-
1536A>G	-	-	-	1.33%	-	-	2.96%	-	-
1619C>T	1.46%	-	-	1.81%	-	-	5.07%	-	-
1661A>G	-	-	-	50.55%	1.41%	-	-	-	-
1842A>G	1.64%	-	-	-	-	-	3.11%	-	-
1883G>A	-	-	-	-	-	-	3.08%	-	-
1888G>A	-	-	-	2.07%	-	-	6.15%	-	-
1889C>A	-	-	-	-	-	-	2.24%	-	-
1900A>G	-	-	-	-	-	-	2.35%	-	-
1944C>T	-	-	-	-	-	-	2.07%	-	-
1977T>C	-	-	-	1.63%	-	-	4.56%	-	-
1978A>G	-	-	-	-	-	-	3.12%	-	-
2000C>T	-	-	-	1.44%	-	-	4.87%	-	-
2056G>A	-	-	-	1.56%	-	-	4.99%	-	-
2059C>T	-	-	-	1.46%	-	-	4.92%	-	-
2080T>C	-	-	-	-	-	-	2.04%	-	-
2143G>A	1.64%	-	-	2.35%	-	-	6.27%	-	-
2162C>T	1.65%	-	-	2.30%	-	-	6.30%	-	-
2168T>C	-	-	-	-	-	-	1.95%	-	-
2523C>T	1.74%	1.29%	1.35%	2.84%	-	1.22%	6.82%	-	-
2541C>A	-	-	-	2.18%	-	-	6.61%	-	-
2557C>T	-	-	-	1.39%	-	-	3.39%	-	-
2572C>T	-	-	-	1.09%	-	-	3.21%	-	-
4048G>A	-	-	-	2.18%	-	-	9.17%	1.33%	-
4104A>G	1.37%	-	-	2.28%	-	-	9.44%	1.44%	-
4312C>T	-	-	-	2.10%	-	-	10.29%	-	-
4318C>T	-	-	-	2.10%	-	-	10.29%	-	-
4456C>T	-	-	-	2.11%	-	-	12.22%	1.84%	-
4736T>C	-	-	-	2.22%	-	-	-	-	-
4856T>C	-	-	-	1.87%	-	-	8.50%	-	-
4904C>T	-	-	-	-	-	-	6.55%	-	-
4914C>T	-	-	-	-	-	-	6.54%	-	-
4940C>T	-	-	-	1.31%	-	-	6.04%	-	-
4958A>G	-	-	-	-	-	-	5.17%	-	-
4991G>A	-	-	-	-	-	-	5.08%	-	-
5041T>C	-	-	-	1.74%	-	-	5.39%	-	-
5147G>A	-	-	-	1.72%	-	-	7.60%	-	-
5320C>T	-	-	-	2.13%	-	-	9.85%	1.19%	-
5351A>G	-	-	-	2.02%	-	-	7.48%	-	-
5385C>T	-	-	-	-	-	-	2.11%	-	-
5387C>T	-	-	-	1.97%	-	-	7.39%	-	-
5426T>C	-	-	-	2.01%	-	-	-	-	-
5471G>A	-	-	-	2.50%	-	-	11.74%	1.47%	-
5474A>G	-	-	-	2.45%	-	-	11.67%	1.43%	-
5493T>C	-	-	-	11.87%	-	-	-	-	-
5498A>G	-	-	-	2.49%	-	-	11.73%	1.40%	-
5580T>C	-	-	-	2.52%	-	-	10.66%	-	-
5821G>A	-	-	-	3.38%	-	-	11.76%	1.91%	-
5840C>T	1.34%	-	-	2.49%	-	-	10.62%	1.32%	-
6023G>A	1.16%	-	-	2.23%	-	-	10.22%	1.22%	-
6221T>C	-	-	-	2.02%	-	-	8.99%	-	-
6242C>T	-	-	-	1.60%	-	-	7.39%	-	-
6266A>C	-	-	-	1.47%	-	-	7.32%	-	-
6299A>G	-	-	-	1.74%	-	-	8.30%	-	-
6366G>A	1.51%	-	-	1.75%	-	-	9.00%	-	-
6383G>A	-	-	-	1.64%	-	-	7.80%	-	-
6405A>G	-	-	-	3.37%	-	-	-	-	-
6410C>T	-	-	-	1.45%	-	-	6.34%	-	-
6452C>T	-	-	-	1.45%	-	-	6.30%	-	-
6483C>T	-	-	-	1.50%	-	-	6.49%	-	-
6512T>C	-	-	-	1.77%	-	-	7.14%	-	-
6542C>T	-	-	-	1.61%	-	-	7.00%	-	-
6569C>A	-	-	-	1.99%	-	-	9.19%	-	-
6641T>C	-	-	-	2.26%	-	-	10.11%	1.18%	-
6935C>T	-	-	-	2.03%	-	-	9.72%	-	-
6938C>T	-	-	-	2.01%	-	-	9.65%	-	-
7146A>G	1.40%	-	-	2.71%	-	-	11.46%	1.37%	-
7195T>C	-	-	-	-	-	-	3.26%	-	-
7197G>A	-	-	-	2.53%	-	-	-	-	-
7216G>A	-	-	-	-	-	-	3.42%	-	-
7232C>T	-	-	-	2.09%	-	-	8.77%	-	-
7256C>T	-	-	1.26%	3.78%	-	1.40%	13.51%	1.70%	-
7286T>C	-	-	-	2.48%	-	-	6.77%	-	-
7299A>G	-	-	-	-	-	-	4.98%	-	-
7316G>A	-	-	-	1.53%	-	-	6.96%	-	-
7325A>G	-	-	-	-	-	-	5.00%	-	-
7337G>A	-	-	-	2.38%	-	-	6.70%	-	-
7364A>G	-	-	-	-	-	-	5.14%	-	-
7473A>G	-	-	-	-	-	-	4.44%	-	-
7521G>A	-	-	-	2.08%	-	-	13.21%	1.56%	-
7559A>G	-	-	-	-	-	-	3.59%	-	-
7571A>G	-	-	-	1.53%	-	-	-	-	-
7610C>T	-	-	-	-	-	-	3.03%	-	-
7650C>T	-	-	-	1.92%	-	-	12.74%	1.39%	-
7702G>A	1.55%	-	-	-	-	-	-	-	-
7705T>C	-	-	-	2.15%	-	-	13.30%	1.58%	-
7757G>A	-	-	-	2.07%	-	-	3.46%	-	-
7810C>T	-	-	-	1.75%	-	-	11.54%	-	-
7861T>C	-	-	-	-	-	-	1.81%	-	-
7868C>T	-	-	-	1.67%	-	-	9.46%	-	-
7891C>T	-	-	-	1.69%	-	-	7.56%	-	-
7912G>A	-	-	-	1.70%	-	-	7.65%	-	-
8021A>G	1.28%	-	-	2.13%	-	-	7.48%	-	-
8065G>A	1.19%	-	-	2.29%	-	-	9.77%	-	-
8167T>C	-	-	-	-	-	-	3.98%	-	-
8203C>T	-	-	-	2.06%	-	-	9.85%	-	-
8392G>A	-	-	-	1.73%	-	-	11.20%	-	-
8455C>T	-	-	-	1.64%	-	-	10.07%	-	-
8461C>T	-	-	-	1.64%	-	-	10.02%	-	-
8503T>C	-	-	-	1.72%	-	-	9.10%	-	-
8537A>G	-	-	-	6.70%	-	-	-	-	-
8545G>A	-	-	-	2.02%	-	-	9.94%	-	-
8655C>T	-	-	-	1.88%	-	-	8.54%	-	-
8677A>C	-	-	-	1.52%	-	-	7.01%	-	-
8701A>G	1.52%	-	-	1.70%	-	-	7.13%	-	-
8718A>G	1.32%	-	-	1.82%	-	-	7.19%	-	-
8943C>T	-	-	-	2.04%	-	-	10.20%	-	-
9060C>A	-	-	-	2.16%	-	-	10.07%	-	-
9075C>T	1.41%	-	-	2.29%	-	-	10.68%	1.30%	-
9168C>T	1.45%	-	-	2.25%	-	-	10.33%	1.26%	-
9254A>G	1.33%	-	-	2.44%	-	-	9.71%	1.28%	-
9325T>C	-	-	-	2.30%	-	-	10.71%	-	-
9329G>C	-	-	-	2.17%	-	-	10.72%	-	-
9434A>G	-	-	-	2.19%	-	-	10.34%	1.31%	-
9629A>G	2.41%	-	-	2.13%	-	-	9.55%	1.39%	-
10945A>G	1.58%	-	-	-	-	-	1.52%	-	-
10978A>G	-	-	-	-	-	-	1.46%	-	-
11016G>A	-	-	-	-	-	-	1.45%	-	-
11083A>G	-	-	-	1.98%	-	-	6.30%	-	-
11097C>T	-	-	-	1.94%	-	-	6.29%	-	-
11147T>C	-	-	-	1.79%	-	-	4.82%	-	-
11176G>A	-	-	-	1.43%	-	-	3.16%	-	-
11197C>T	-	-	-	1.40%	-	-	3.16%	-	-
11233T>C	-	-	-	-	-	-	1.65%	-	-
11527C>T	-	-	-	2.06%	-	-	6.65%	-	-
11557A>G	-	-	-	2.15%	-	-	6.67%	-	-
11590A>G	-	-	-	2.02%	-	-	6.73%	-	-
11662T>C	-	-	-	2.07%	-	-	6.85%	-	-
11852G>A	-	-	-	-	-	-	3.14%	-	-
11857C>T	-	-	-	-	-	-	3.00%	-	-
11887G>A	-	-	-	1.76%	-	-	5.42%	-	-
11914G>A	1.89%	1.43%	1.47%	1.80%	-	-	5.43%	-	-
11963G>A	-	-	-	1.65%	-	-	5.75%	-	-
12007G>A	-	-	-	1.67%	-	-	5.85%	-	-
12013A>G	-	-	-	1.76%	-	-	5.87%	-	-
12018C>G	-	-	-	-	-	-	3.75%	-	-
12561G>A	-	-	-	-	-	-	3.71%	-	-
12684G>A	-	-	-	2.26%	-	-	10.52%	1.60%	-
12705C>T	-	-	-	2.26%	-	-	10.51%	1.63%	-
13062A>G	-	-	-	2.21%	-	-	10.83%	1.59%	-
13095T>C	1.96%	-	-	2.38%	-	-	9.43%	1.64%	-
13105A>G	1.80%	-	-	2.32%	-	-	9.41%	1.55%	-
13242A>G	-	-	-	1.51%	-	-	2.29%	-	-
13260T>C	-	-	-	1.59%	-	-	2.36%	-	-
13272C>T	-	-	-	1.30%	-	-	2.00%	-	-
13281T>C	-	-	-	1.36%	-	-	2.08%	-	-
13359G>A	-	-	-	-	-	-	3.54%	-	-
13368G>A	-	-	-	-	-	-	3.51%	-	-
13386T>C	1.28%	-	-	1.78%	-	-	5.13%	-	-
13440C>T	-	-	-	1.61%	-	-	4.89%	-	-
13466G>A	-	-	-	1.55%	-	-	4.70%	-	-
13476A>G	-	-	-	1.55%	-	-	4.65%	-	-
13488T>C	-	-	-	1.76%	-	-	4.99%	-	-
13563A>G	-	-	-	-	-	-	6.73%	-	-
13581T>C	-	-	-	-	-	-	6.73%	-	-
14149C>T	-	-	-	-	-	-	3.11%	-	-
14170A>T	-	-	-	-	-	-	3.15%	-	-
14172T>G	-	-	-	1.58%	-	-	4.74%	-	-
14182T>C	-	-	-	1.74%	-	-	4.90%	-	-
14950C>T	-	-	-	-	-	-	3.74%	-	-
14956T>C	-	-	-	1.21%	-	-	3.85%	-	-
14969T>C	-	-	-	-	-	-	3.72%	-	-
15191T>C	1.84%	1.50%	1.69%	-	-	-	-	-	-
15301G>A	2.80%	1.92%	2.19%	-	-	-	-	-	-
16129G>A	-	-	-	9.25%	-	-	-	-	-
16182A>C	-	-	-	-	-	-	26.73%	24.66%	23.82%
16183A>C	-	-	-	-	-	-	60.43%	58.92%	60.06%
16444C>T	-	-	-	1.42%	-	-	-	-	-
16496G>A	-	-	-	1.50%	-	-	-	-	-
16527C>T	-	-	-	2.08%	-	-	-	-	-

Interestingly, there was a complete lack of heteroplasmy SNPs in the 30% to 50% frequency range. However, when the additional 222 samples were analyzed, there were 13 heteroplasmic SNPs in the 30–39% range; 9 heteroplasmic SNPs in the 40–49% range and 10 heteroplasmic SNPs in the 50–59% range. In comparison, there were more than 2,200 SNPs in the 90–99% range. The low numbers of heteroplasmic SNPs in the 30% to 50% range suggests that those heteroplasmic SNPs may somehow be eliminated from the mtDNA pool.

There were 10 heteroplasmic SNPs that were found only in the blood and not in [RPE+choroid] or Retina samples; (m.1292A>G, m.1619C>T, m.2143G>A, m.2162C>T, m.6366G>A, m.8021A>G, m.8065G>A, m.8701A>G, m.8718A>G, and m.13386T>C).

**Table 6** shows 23 heteroplasmy ‘hot-spot’ SNPs that are present in each of the three Subjects. The ‘hot-spots’ were defined as heteroplasmy SNPs found in at least two out of the three subjects. There are 13 heteroplasmy SNPs found in all three subjects (pink boxes). Ten heteroplasmy SNPs were found in 2 out of the 3 subjects (blue boxes). The majority of ‘hot-spot’ SNPs were found in the blood samples for Subjects #1 and #2. Subject #3 showed the ‘hot-spot’ SNPs in both blood and [RPE+choroid]. In total there was only 1 ‘hot-spot’ SNP found in the retinal tissue of the three subjects (m.7256C>T). **Table 7** shows three of the ‘hot-spot’ SNPs were non-synonymous (amino acid change): m.5320C>T, Thr284Ile; m.7146A>G, Thr415Ala and m.13105A>G, Ile257Val. Thirteen of the ‘hot-spot’ SNPs were synonymous and resulted in no amino acid change. As evaluated by the website www.hmtvar.uniba.it, four of the ‘hot-spot’ SNPs have been associated with pathogenicity (m.1120C>T, m.1556C>T, m.9254A>G and m.12705 C>T). Three of the SNPs have HmtVar Predictions of Polymorphic Disease (m.5840C>T, <0.35; m.7146A>G, < 0.43; and m.13105 >G, < 0.42). Many of the heteroplasmy SNPs had PhyloP scores representing the conservation of the variant. The positive value is a conserved site (e.g., m.5498A>G, Syn:Met343) while the negative numbers are associated with fast-evolving sites, some of which are synonymous (e.g., m.5474A>G, Syn:Leu335) but others are non-synonymous (m.5320C>T, Thr284Ile).

**Table 6:** Top 23 heteroplasmy SNPs: ‘Hot-spots’.

	Frequency				Frequency				Frequency		
	Subject 1				Subject 2				Subject 3		
SNP Change	Blood	[RPE+choroid]	Retina	SNP Change	Blood	[RPE+choroid]	Retina	SNP Change	Blood	[RPE+choroid]	Retina
1120C>T	1.67%	none	none	1120C>T	3.23%	none	1.24%	1120C>T	8.92%	1.40%	none
1284T>C	1.90%	none	none	1284T>C	2.73%	none	1.16%	1284T>C	8.20%	none	none
1556C>T	2.14%	none	none	1556C>T	3.68%	none	1.38%	1556C>T	10.52%	1.69%	none
2523C>T	1.74%	1.29%	1.35%	2523C>T	2.84%	none	1.22%	2523C>T	6.82%	none	none
4048G>A	1.37%	none	none	4048G>A	2.28%	none	none	4048G>A	9.17%	1.33%	none
4104A>G	1.37%	none	none	4104A>G	2.28%	none	none	4104A>G	9.44%	1.44%	none
4556C>T	none	none	none	4556C>T	2.11%	none	none	4556C>T	12.22%	1.84%	none
5320C>T	none	none	none	5320C>T	2.13%	none	none	5320C>T	9.85%	1.19%	none
5471G>A	none	none	none	5471G>A	2.50%	none	none	5471G>A	11.74%	1.47%	none
5474A>G	none	none	none	5474A>G	2.45%	none	none	5474A>G	11.74%	1.47%	none
5498A>G	none	none	none	5498A>G	2.49%	none	none	5498A>G	11.73%	1.40%	none
5840C>T	1.34%	none	none	5840C>T	2.49%	none	none	5840C>T	10.62%	1.32%	none
6023G>A	1.16%	none	none	6023G>A	2.23%	none	none	6023G>A	10.22%	1.22%	none
7146A>G	1.40%	none	none	7146A>G	none	none	none	7146A>G	11.46%	1.37%	none
7256C>T	none	none	1.26%	7256C>T	3.78%	none	1.40%	7256C>T	13.51%	1.70%	none
9075C>T	1.41%	none	none	9075C>T	2.29%	none	none	9075C>T	10.68%	1.30%	none
9168C>T	1.45%	none	none	9168C>T	none	none	none	9168C>T	10.33%	1.26%	none
9254A>G	1.33%	none	none	9254A>G	none	none	none	9254A>G	9.71%	1.28%	none
9629A>G	2.41%	none	none	9629A>G	none	none	none	9629A>G	9.55%	1.39%	none
11914G>A	1.89%	1.43%	1.47%	11914G>A	1.80%	none	none	11914G>A	5.43%	none	none
12705C>T	99.12%	99.59%	99.71%	12705C>T	2.26%	none	none	12705C>T	10.51%	1.63%	none
13095T>C	1.96%	none	none	13095T>C	2.38%	none	none	13095T>C	9.43%	1.64%	none
13105A>G	1.80%	none	none	13105A>G	2.32%	none	none	13105A>G	9.41%	1.55%	none

Heteroplasmy present in all three individuals

Heteroplasmy present in two out of the three individuals

**Table 7:** Characterization and pathogenicity of 15 heteroplasmy ‘Hot-spots’.

SNP Change	Locus Type	Locus	AA Change	Codon Position	rs Number	Pathogenicity
1120 C>T	rRNA	MT-RNR1	n/a	n/a	rs727505171	Progressive Encephalopathy / PEO, myopathy
1284 T>C	rRNA	MT-RNR1	n/a	n/a	n/a	n/a
1556 C>T	rRNA	MT-RNR1	n/a	n/a	n/a	Sensorineural Hearing Loss
4104 A>G	Coding	MT-ND1	Syn:Leu266	3.0	rs1117205	n/a
4556C>T	Coding	MT-ND2	Syn:Thr29	3.0	n/a	PhyloP score -0.48 (Negative, fast-evolving site)
5320C>T	Coding	MT-ND2	Thr284Ile	2.0	n/a	PhyloP score -3.21
5471G>A	Coding	MT-ND2	Syn:Thr334	3.0	n/a	PhyloP score -10.63
5474A>G	Coding	MT-ND2	Syn:Leu335	3.0	n/a	PhyloP score -12.09
5498A>G	Coding	MT-ND2	Syn:Met343	3.0	n/a	PhyloP score 6.56 (Positive, conserved site)
5840 C>T	tRNA	MT-TY	n/a	n/a	n/a	HmtVar Prediction: Likely Polymorphic
						Disease score <0.35
6023 G>A	Coding	MT-CO1	Syn:Glu40	3.0	n/a	n/a
7146 A>G	Coding	MT-CO1	Thr415Ala	1.0	rs372136420	HmtVar Prediction: Polymorphic
						Disease score <0.43
7256 C>T	Coding	MT-CO1	Syn:Asn451	3.0	n/a	n/a
9075 C>T	Coding	MT-ATP6	Syn:Thr183	3.0	n/a	n/a
9168 C>T	Coding	MT-ATP6	Syn:Phe214	3.0	n/a	n/a
9254 A>G	Coding	MT-CO3	Syn:Trp16	3.0	rs386829072	Pancreatic cancer cell line
9629 A>G	Coding	MT-CO3	Syn:Gly141	3.0	n/a	n/a
11914G>A	Coding	MT-ND4	Syn:Thr385	3.0	rs2853496	n/a
12705 C>T	Coding	MT-ND5	Syn:Ile123	3.0	rs193302956	Prostate tumor
13095 T>C	Coding	MT-ND5	Syn:Val253	3.0	rs28477492	n/a
13105 A>G	Coding	MT-ND5	Ile257Val	1.0	rs2853501	HmtVar Prediction: Polymorphic
						Disease score < 0.43

n/a: not available.

Syn: synonymous.

The frequency of the 23 heteroplasmy ‘hot-spot’ SNP changes were analyzed in 222 additional blood samples; Old-AMD (n = 73), Old-NL (n = 66), and Young-NL (n = 83) (**Table 8**). We showed 4 ‘hot-spot’ heteroplasmy SNPs that were highly conserved with 97% to 100% frequency in the Young-NL, Old-NL and Old-AMD samples (m.1120C>T; m.1284T>C; m.1556C>T; m.7256A>G). **Tables 8 and 9** show that the m.4101A>G heteroplasmy declined with age (Young-NL versus Old-NL, Odds ratio of 26.24, P = 1.1E-5 and Young-NL versus Old-AMD, Odds ratio 26.84, P = 8.0E-6). Seven other heteroplasmy SNPs were decreased in Old-NL compared to Young-NL (m.5320C>T, P = 0.0029; m.5471G>A, P = 6.5E-5; m.5474A>G, P = 8.0E-6; m.5498A>G, P = 4.6E-6; m.9168C>T, P = 0.0204; m.9254A>G, P = 0.0029 and m.9629A>G, P = 0.0057) but all were synonymous amino acid changes except m.5320C>T which was non-synonymous (Thr284Ile). However, the m.13105A>G variant causing a non-synonymous amino acid change (Ile257Val) was higher in the Old-AMD compared to the Old-NL (Odds ratio 0.365, P = 0.0175) and the Young-NL (Odds ratio 0.348, P = 0.009). These results demonstrate that ‘hot-spot’ heteroplasmy SNPs were found in Old-AMD, Old-NL, and Young-NL subjects, which may influence the bioenergetic profile of the cells.

**Table 8:** mtDNA heteroplasmy 23 hot-spots found in blood of 222 subjects.

SNP Change	Total SNPs (n = 222)	Young Normal (n = 83)	Older Normal (n = 66)	Older AMD (n = 73)
1120 C>T	219	82 (98.8%)	64 (97%)	73 (100%)
1284 T>C	218	82 (98.8%)	64 (97%)	72 (98.6%)
1556 C>T	218	82 (98.8%)	64 (97%)	72 (98.6%)
4104 A>G	187	82 (98.8%)	50 (75.8%)	55 (75.3%)
4556C>T	0	0	0	0
5320C>T	185	78 (93.4%)	51 (77.3%)	56 (76.7%)
5471G>A	170	78 (93.4%)	44 (66.7%)	48 (65.8%)
5474A>G	170	78 (93.4%)	44 (66.7%)	48 (65.8%)
5498A>G	156	74 (89.2%)	37 (56.1%)	45 (61.6%)
5840 C>T	194	77 (92.8%)	54 (81.8%)	63 (86.3%)
6023 G>A	198	78 (94%)	55 (83.3%)	65 (89%)
7146 A>G	197	78 (94%)	56 (81.8%)	63 (86.3%)
7256 C>T	216	81 (97.6%)	64 (97%)	71 (97.2%)
9075 C>T	198	78 (94%)	55 (83.3%)	65 (89%)
9168 C>T	196	78 (94%)	54 (81.8%)	64 (87.7%)
9254 A>G	192	78 (94%)	51 (77.3%)	63 (86.3%)
9629 A>G	192	78 (94%)	52 (77.3%)	62 (84.9%)
11914G>A	188	75 (90.4%)	51 (77.3%)	62 (84.9%)
12705 C>T	182	69 (83.1%)	50 (75.8%)	63 (86.3%)
13095 T>C	165	56 (67.5%)	47 (71.2%)	62 (84.9%)
13105 A>G	166	57 (68.7%)	46 (69.7%)	63 (86.3%)

**Table 9:** List of heteroplasmy SNPs comparing young-NL, older-NL and AMD groups.

	Young-NL vs Old-NL Odds ratio; P value	Young-NL vs AMD	Old NL vs AMD	Amino Acid Change
**m.4101A>G**	26.24; p = 0.0018	26.84; p = 0.0016	NS	No
**m.5320C>T**	4.588; p = 0.0029	4.73; p = 0.0019	NS	Thr284Ile
**m.5471G>A**	5.8; p = 6.5E-5	7.16; p = 4.0E-6	NS	No
**m.5474A>G**	8.34; p = 8.0E-6	8.13; p = 8.0E-6	NS	No
**m.5498A>G**	6.44; p = 4.6E-6	5.12; p = 5.6E-5	NS	No
**m.9168C>T**	3.466; p = 0.0204	2.193; p = 0.169	NS	No
**m.9254A<G**	4.588; p = 0.0029	2.476; p = 0.105	NS	No
**m.9629A>G**	4.2; p = 0.0057	2.76; p = 0.063	NS	No
**m.13095T>C**	0.838; p = 0.623	0.367; p = 0.011	0.439; p = 0.049	No
**m.13105A>G**	0.953; p = 0.018	0.348; p = 0.009	0.365; p = 0.017	Ile257Val

### Transversion versus transition mutations

The mutations within the mtDNA can be classified as those that are transitions (purine to purine (A, G) or pyrimidine to pyrimidine (T, C)) and are indicative of replication errors. The transversion mutations are purine to pyrimidine or vice versa and are associated with higher levels of oxidative damage [38]. When comparing the blood, [RPE+choroid] and retinal heteroplasmy SNPs from the 3 subjects, we found few transversion mutations (**Table 10**). Subject #1 showed only 1 out of 35 heteroplasmy SNPs in the blood was a transversion mutation (567A>C). The rest were transition changes (34/35). Subject #2 showed 4.4% of the heteroplasmy SNPs (6/135) in the blood were a result of transversion mutations while the rest were transition mutations (129/135). There were no transversion mutations in the [RPE+choroid] or retinal mtDNA for Subjects #1 and #2. Subject #3 had 8.48% (14/165) transversion mutations in the blood; 6.25% (2/32) in the [RPE+choroid] and 66.6% (2/3) in the retina. Again, the majority of heteroplasmy SNPs in Subject #3 were transition mutations. Our findings suggest that the heteroplasmic changes may be more related to replication errors (transitions) rather than oxidative damage, which lead to transversion mutations.

**Table 10:** Transversion versus transition mutations in mtDNA of each subject.

**A. Subject 1**			
**Percentage Frequency**			
**Transversion Heteroplasmy Mutations**	**Blood**	**[RPE+choroid]**	**Retina**
567A>C	3.36%	0	0
**Total # Heteroplasmy SNPs (Transversion Plus Transition)**	35	8	9
**Transversion mutations**	1	0	0
**Transitions mutations**	34	8	9
**B. Subject 2**			
**Percentage Frequency**			
**Transversion Heteroplasmy Mutations**	**Blood**	**[RPE+choroid]**	**Retina**
2541C>A	2.18%	0	0
6569C>A	1.99%	0	0
8677A>C	1.52%	0	0
9060C>A	2.16%	0	0
9329G>C	2.17%	0	0
14172T>G	1.58%	0	0
**Total # Heteroplasmy SNPs (Transversion Plus Transition)**	135	5	8
**Transversions mutations**	6	0	0
**Transitions mutations**	129	5	8
**C. Subject 3**			
**Percentage Frequency**			
**Transversion Heteroplasmy Mutations**	**Blood**	**[RPE+choroid]**	**Retina**
825T>A	7.5%	0	0
1123C>G	3.66%	0	0
1889C>A	2.24%	0	0
2541C>A	6.61%	0	0
6266A>C	7.32%	0	0
6569C>A	9.19%	0	0
8677A>C	7.01%	0	0
9060C>A	10.07%	0	0
9329G>C	10.72%	0	0
12018C>G	3.75%	0	0
14170A>T	3.15%	0	0
14172T>G	4.74%	0	0
16182A>C	26.73%	24.66%	23.82%
16183A>C	60.43%	58.92%	60.06%
**Total # Heteroplasmy SNPs (Transversion Plus Transition)**	165	32	3
**Transversions mutations**	14	2	2
**Transitions mutations**	151	30	1

## Discussion

Rapid next generation sequencing (NGS) is a well-established method to evaluate disease-associated changes to the nuclear genome but evaluation of the mtDNA has not achieved the same degree of analyses. The Illumina HumanHap 550 chip has been used to identify the mtDNA haplogroups and their association with autism spectrums [39]. The SNP coverage allows for identification of the major nodal haplogroups but the detailed haplogroup subsets cannot be resolved with the information generated from this chip. The custom-made Illumina HumanCoreExome array has been used to investigate large cohorts of AMD subjects to identify nuclear and select mtDNA variants associated with disease phenotype [40]. However, this array possesses only 265 mtDNA SNP variants, making it difficult to diagnose rare mitochondrial variants or heteroplasmy SNPs that contribute to the disease. The exome sequencing combined with rapid mitochondrial genome sequencing has allowed identification of mtDNA deletions in newborn infants [41]. The methods used involved sequencing on a MiSeq instrument using v2 chemistry (Illumina) that identified single nucleotide variant heteroplasmy greater than 3% and large indels (greater than1 kb) with heteroplasmy >30%. It has been generally felt that the exome sequencing does not deliver adequate coverage of the mtDNA and a new diagnostic approach is desirable. More recently, Trounce et al., used next-generation sequencing (NGS) to identify the mtDNA haplogroups and genetic burden of rare variants from blood of patients with open angle glaucoma [42]. However, they did not analyze for low frequency heteroplasmy SNPs. A different approach used for forensic identification of fragmented mtDNA is a whole genome panel that is a 2 pool multiplex assay of 81 primer pairs with minimal overlap in each pool (Precision ID mtDNA Whole Genome Panel, Thermo Fisher Scientific). This system has been designed for analyses of degraded DNA and is not typically used for research or disease association.

Using the NGS technique, we demonstrated that within an individual, the blood, [RPE+choroid] and retina contained similar numbers of homoplasmy SNPs representing the inherited germline mtDNA genome (haplogroups). This was not surprising since the mtDNA haplogroups patterns are maternally inherited and should be consistent within the tissues and within siblings that have the same mother. Based upon the SNP pattern, Subject #1 was I1a1b haplogroup, Subject #2 was H7b and Subject #3 was U2e2a1. The homoplasmy SNPs with greater than 89% frequency were haplogroup defining SNPs. Subject #2 was a H7b haplogroup and the numbers of homoplasmy SNP in blood-[RPE+choroid] and retina were only 3, which is to be expected because the sequence analyses are compared to the Cambridge reference sequence that is also H haplogroup (NC_012920). It has been recognized for some time that organs from an individual can contain different mtDNA heteroplasmy levels, which may contribute to the selectivity of involvement of one organ versus another in mitochondrial diseases damage [43].

The blood for each of the subjects showed the largest numbers of total SNPs and the greatest variability between subjects. Interestingly, the two wet AMD subjects (#2 and #3) had higher numbers of SNPs in the blood (144 and 205 SNPs, respectively) while the subject with dry AMD (#1) showed the lowest SNP numbers in the blood (71 SNPs). While intriguing that blood mtDNA SNP numbers might be associated with disease phenotype (wet versus dry), additional studies with larger numbers of AMD patients need to be performed to determine a relationship.

It has been demonstrated that retinas, which are very metabolically active, can have high degree of oxidative damage with increasing age, and the mtDNA can acquire heteroplasmy when exposed to oxidative stress environments. Ramos et al., have shown that more than 61% of individuals have some frequency of mtDNA heteroplasmy and if it happens at a stable site in the mtDNA genome, then dysfunction may occur [44]. At the beginning of the study, we had speculated that the mtDNA heteroplasmy would be elevated in the neuroretina and [RPE+choroid] (RPE cells plus choroid) as compared to the blood. Using our novel NGS methodology for deep sequencing to identify SNPs with 40% or less heteroplasmy, we were surprised to find significantly more mtDNA heteroplasmy SNPs in blood compared to retina and [RPE+choroid] from the same subject as well as across 3 subjects, suggesting significant protection in the retina/[RPE+choroid] as compared to blood. When values from the 3 subjects were combined, the blood had significantly greater numbers of heteroplasmy SNPs than the retina alone (95% higher, P < 0.05) or retina/[RPE+choroid] combined (89% higher, P = 0.008). While the mechanisms are not known, one can speculate that since the blood cells have rapid turnover, the higher degree of heteroplasmy can be tolerated in the cell. In contrast, the cells of the neuroretina and [RPE plus choroid] have little turnover so only low levels of mtDNA heteroplasmy are acceptable. It suggests that these tissues have a yet to be identified mechanism to keep the heteroplasmy levels low.

The mtDNA mutations that accumulate with age in the RPE and retina can be generated through transitions (purine to purine (A,G) or pyrimidine to pyrimidine (C,T)) changes, which occurs by replication errors or alternatively through transversion changes (purine to pyrimidine or vice versa) that are associated with response to oxidative stress/damage. The tissues examined in this study were from individuals who were 93 years of age (Subject #1), 88 years of age (Subject #2) and 90 years of age (Subject #3) so we speculated that the majority of heteroplasmy SNPs would have originated via transversion changes. Surprisingly, in blood the heteroplasmy SNPs of Subject #1 had only 2.86% and Subject #2 had 4.4% transversion changes and none were present in the [RPE+choroid] or retina. Subject #3 had 8.48% transversion changes in the blood, 6.25% in the [RPE+choroid] and 66.6% (2 out of 3 SNPs) in the retina. This suggests that, in spite, of the increased age of the tissues, the majority of the heteroplasmy SNPs occurred because of transition changes due to replication error rather than resulting from oxidative damage. This is in agreement with studies of *Drosophila melanogaster* that compared young versus old fruit flies and reported only a small fraction of the mutations were due to transversion changes but most were a result of errors in mtDNA replication [38]. Zsurka et al. have suggested that the accumulation of somatic mtDNA mutations may be related more to the fidelity of mtDNA polymerase γ (POLG) rather than oxidative damage [45]. It may also be disease dependent, as diabetics have higher somatic transversion mutation rates compared to controls [46]. NGS of chicken mtDNA genomes showed that 83.7% of the heteroplasmic mutations were nucleotide transitions, while the remainder were transversions detected at low frequency [47]. In addition, the majority of heteroplasmic SNPs were found in the D-Loop. Examination of 10 different tissues from the same individual chicken showed 80% of the SNP heteroplasmy was found in only a single tissue type and the occurrence of heteroplasmic SNPs decreased in subsequent generations (F0 to F1).

The relatively low level of transversions in the heteroplasmic SNPs suggests they may disadvantageous to mtDNA and not tolerated when found in high frequencies. One study has suggested that the heteroplasmic transversion SNP, *mt*.*T5718G*, may predict changes to secondary structure of RNA [47]. Interestingly, in our study none of the ‘hotspot’ SNPs were transversion mutations. The functional consequences of the heteroplasmic SNPs is still controversial. Lu et al. sequenced the mtDNA of poultry and showed that mtDNA heteroplasmy in the MT-ND2 gene is associated with pectoral muscle fat content [48]. Normally there is rapid accumulation of heteroplasmic mtDNA SNPs in germline and somatic cells but there are as of yet unknown mechanisms to remove and regulate the these variants to minimize their potential detrimental effects [49]. Ziada et al. reported increased transversion and transition mutations associated with aging, with the latter being more common [50]. While the mitochondria are a major source of endogenous ROS that can damage proteins, lipids and DNA, the contribution of the oxidative damage to mtDNA heteroplasmic mutations needs to be further investigated.

It has been reported that pathogenicity affecting cellular function is achieved at 85–90% heteroplasmy of a point mutation [1, 51, 52]. In our NGS results, the vast majority of heteroplasmy ranged from 1.16% to 13.3% frequency. The two exceptions were the m.16182A>C heteroplasmy SNPs at 26.73% frequency and m.16183A>C at 60.43% frequency. Pichard et al. reported that individuals with increasing levels of the m.3243A>G heteroplasmy showed different clinical diseases (diabetes at 10–30% mutations; encephalomyopathies at 50–90% mutations and perinatal lethality at 90–100% mutations) [53]. Furthermore, cybrid cells showed variable functional and structural features depending upon the levels of m.3243A>G heteroplasmy levels. It is not clear how cells maintain a relatively narrow range of heteroplasmy and whether mitochondria and cells are eliminated once the 30% threshold is reached. Additional studies will be necessary to clarify the mechanisms.

Using a transmitochondrial cybrid model, Kopinski et al showed that higher levels of m.3243A>G heteroplasmy led to lower levels of acetyl-CoA and histone H4 acetylation, while mid-levels of heteroplasmy increased alpha-ketoglutarate levels and decreased histone H3 methylation [54]. The degree of mtDNA heteroplasmy also affects the ratio of mitochondrial NAD+/NADH, which correlates with acetylation of nuclear histones. Therefore, the influence in disease processes may occur because the mtDNA heteroplasmic SNPs alter the cellular epigenetic status leading to changes in downstream gene expression patterns. Analyses of mtDNA from 1363 post mortem brains revealed approximately 32% of the tissues had high frequency of heteroplasmy mtDNA variants but there was no association of a single heteroplasmic SNP with brain diseases or aging [55]. These findings support the need for reliable, efficient deep NGS methods to better understand the correlation with mtDNA variants, including heteroplasmic SNPs, and disease processes. To our knowledge this is the first study to identified ‘hot-spots’ of heteroplasmy SNP changes (frequency ranging from 1.16% to 13.51%) in human mitochondria. Of the 23 SNPs, 13 of the heteroplasmic SNPs were found in all three subjects and 10 were in two of the three subjects. The blood was the most common tissue to possess the ‘hot-spot’ heteroplasmy SNPs. There were two non-synonymous ‘hot-spots’ that resulted in amino acid changes in the MT-CO2 and MT-ND5 regions and both were listed to have prediction polymorphic diseases scores. To determine if these 23 ‘hot-spots’ were present in other subjects, an addition 222 blood mtDNA samples were examined. There were Old-AMD (n = 73), Old-NL (n = 66), and Young-NL (n = 83) subjects. Four of the ‘hot-spot’ heteroplasmy SNPs (m.1120C>T; m.1284T>C; m.1556C>T; m.7256A>G) were found in 97% to 100% of the Young-NL, Old-NL and Old-AMD samples, indicating that these sites were highly susceptible to transition mutations. Three of the ‘hot-spot’ SNPs were in the MT-RNR1 (rRNA) region, while the fourth (m.7256C>T) caused a synonymous amino acid change. One can speculate that increased ‘hot-spot’ mutations in MT-RNR1 region may alter the levels of the Mitochondrial Derived Peptides (MDPs) in some individuals. Hopkins et al showed that mutational ‘hot-spots’ were associated with aggressive prostate cancer [56]. Others have reposted that tRNA ‘hot-spots’ found in muscle were linked to mitochondrial myopathies and exercise intolerance [57]. Some MT-tRNA ‘hot-spot’ mutations (m.1659C>T, m.5650G>A and m.15975C>T) have been reported to be pathogenic in hepatocellular carcinomas [58].

In the Young-NL group, 98.8% of the subjects had heteroplasmy in the m.4104A>G SNP. In contrast, the Old-NL (75.8%) and AMD (75.3%) had significantly fewer individuals with the m.4104A>G heteroplasmy SNP. The heteroplasmy levels in m.5471G>A; m.5498A>G; m.9168C>T, m.9254A>G and m.9629A>G were also lower in the Old-NL compared to Young-NL samples. However, each of these are synonymous, not causing an amino acid change. The m.5320C>T heteroplasmy was lower in Old-NL and AMD compared to Young-NL and results in a Thr284Ile amino acid change in the MT-ND2 gene. One can speculate that the amino acid change might decrease OXPHOS efficiency in the aging/AMD groups, but additional studies are required for verification. The old-AMD subjects had increased levels of m.13105A>G that causes an amino acid change (Ile257Val) in MT-ND5 compared to both the Old-NL and Young-NL. The m.13105A>G variant has a HmtVar Prediction score < 0.43 suggesting that it may be pathogenic. The functional significance of this SNP change within the AMD groups is not clear currently but deserves further study.

Generation of heteroplasmic mice through cross-breeding demonstrated offspring with impaired learning, diminished physical capacity and behavioral differences [59]. These results suggest heteroplasmic SNPs can have deleterious physiological effects on the individual and may play a role in disease-associated pathology. The importance of heteroplasmic SNPs in human diseases are an unexplored field, in part because the sequencing techniques for mtDNA have not kept up with those for the nuclear genome. Terluk et al reported that mtDNA from AMD subjects had higher levels of DNA lesions [32], which might slow down or block the progression of a thermostable DNA polymerase and prevent complete product synthesis. To counter this situation, our method uses the dual strand sequencing approach which is to eliminate the “false” identification of these lesions as variants that have functional significance. When there are lesions (generally on one strand only), they will be eliminated because they will not get coverage from both strands. It’s also important to keep in mind that the results of sequencing are from a mixture of cell population, and we are not looking at a single mitochondrial sequencing. The data from each strand can be analyzed to identify these lesions but that is not the aim of this study. The aim of this study is to identify “true” low frequency somatic Single nucleotide variants (SNV’s) that have functional significance and compare it within different tissues of the same individual.

The mechanism behind developing ‘hot-spots’ is not understood. Villagran and Miller have used computational DNA hole spectroscopy to show that a positive charge site is created when an electron is removed and this may trigger mtDNA replication base pair mismatching and possible disease associated mutations [60, 61]. Further work is needed to clarify the importance of heteroplasmy SNP ‘hot-spots’ in aging and diseases.

In summary, an individual has identical homoplasmy SNPs representing the mtDNA haplogroup within the blood, [RPE+choroid] and retina tissues. Using NGS methodology, larger numbers of heteroplasmy SNPs were identified in blood compared to retina and [RPE+choroid], suggesting significant protection in these tissues. The majority of heteroplasmy SNPs were transition mutations (purine to purine or pyrimidine to pyrimidine), which suggests that accumulation of heteroplasmy may be occurring through replication errors rather than oxidative damage (transversion mutations, purine to pyrimidine or vice versa). Further NGS studies will be required to more completely understand the significance of the genomic changes of mtDNA associated with aging, diseases, including AMD, and tissue specificity within a single individual.

## Supporting information

S1 Table (XLSX)
